# Enhancing geohazard-based natural hazards triggering technological accidents’ preparedness: A policy framework in Indonesian context

**DOI:** 10.4102/jamba.v18i1.2062

**Published:** 2026-08-28

**Authors:** Andi E. Sakya, Nuraini R. Hanifa, Achmad F. Shomim, Putri N. Ratna, Aulia Oktaviani, Mizan B.F. Bisri, Ainun Z. Noor

**Affiliations:** 1 Research Centre for Geological Disaster, National Research and Innovation Agency (BRIN), Bandung, Indonesia; 2 Asian Disaster Reduction Centre, Kobe University, Kobe, Japan; 3 Directorate of Repository, Multimedia, and Scientific Publishing, National Research and Innovation Agency (BRIN), South Tangerang, Indonesia

**Keywords:** NATECH, disaster risk governance, geohazards, industrial resilience, Indonesia, policy framework

## Abstract

Indonesia’s rapid industrialisation and high exposure to destructive geohazards (earthquakes, tsunamis, landslides) create an escalating and under-addressed risk of natural hazards triggering technological accidents (NATECH) and systemic industrial failures. This convergence necessitates a robust governance framework, yet current national policies remain fragmented and reactive, lacking explicit provisions for NATECH risk integration. This study delivers an integrated evidence base to diagnose critical gaps in Indonesia’s NATECH governance. Using a mixed-method approach, combining a bibliometric review of 47 publications (2007–2025), geospatial mapping and regulatory assessment, the findings reveal minimal research attention, weak cross-sectoral coordination and a policy vacuum within key disaster and environmental regulations. Specifically, bibliometric mapping using VOSviewer is employed to identify research trends and knowledge gaps, while a bibliometric literature review guided by the Preferred Reporting Items for Systematic reviews and Meta-Analyses (PRISMA) framework ensures transparency and reproducibility in the article selection process. Geospatial analysis further confirms that numerous special economic zone (SEZ) facilities are critically located within high-risk seismic and tsunami zones. We argue that the systemic lack of NATECH mainstreaming necessitates fundamental regulatory reform. We propose a geohazard-based NATECH preparedness policy framework structured around three pillars: (1) establishing a national NATECH database and detailed risk mapping; (2) mandating standardised industrial preparedness schemes based on global best practices; and (3) enacting binding regulatory mechanisms through the harmonisation of laws across key sectors.

**Contribution:**

This framework provides a crucial roadmap for enhancing industrial resilience and reducing cascading risk in one of the world’s most geologically active regions.

## Introduction

Technological accidents triggered by natural hazards or natural hazards triggering technological accidents (NATECH) are a growing global concern with potentially severe consequences. These events occur when natural hazards, such as earthquakes, tsunamis, floods or extreme weather events, disrupt industrial facilities, often leading to hazardous material releases, fires or explosions that threaten human safety and the environment (Cruz & Krausmann 2009; Showalter & Myers 1994). The 2011 Fukushima nuclear disaster following the M9.0 earthquakes and tsunami in Japan highlighted the need to address the complex interplay between natural and technological hazards, underscoring the challenges NATECH events pose to environmental, public health and economic resilience (Cruz & Suarez-Paba 2019; Krausmann, Cruz & Salzano 2017; Krausmann, Girgin & Necci 2019; Lipscy, Kushida & Incerti 2013; UNDRR-APSTAAG 2020).

Global examples, such as Hurricane Katrina in 2005 and the Fukushima disaster in 2011, illustrate how NATECH events can devastate infrastructure, disrupt supply chains and hinder recovery efforts (Knabb, Rhome & Brown 2023; Cruz & Krausmann 2013). Recent studies further highlight that climate change is amplifying the frequency, intensity and spatial extent of hazard-triggered technological risks, particularly in rapidly industrialising regions (Luo et al. 2024; Pilone et al. 2021; UNDRR 2022) Furthermore, globally, most NATECH research is concentrated in high-income and industrialised countries, especially Japan, Italy, and Turkey (Cruz & Krausmann 2013; Cruz & Okada 2008; Girgin, Necci & Krausmann 2019).

Positioned at the Ring of Fire, Indonesia faces a compounding risk of NATECH catastrophes; here, the intersection of frequent seismic, tsunami and volcanic activities with rapid, dense industrialisation has created a critical vulnerability gap that demands immediate systemic intervention (BNPB 2024; CFE-DM 2025; Lestari et al. 2021). Geohazards, although less frequent, cause more significant economic damage, exacerbated by rapid industrial development in high-risk zones (Meilano 2020). Table 1 highlights a striking contrast: geohazards, although less frequent, inflict far greater damage per event than other common hazards (Meilano et al. 2022). This vulnerability is further compounded by the rapid and often unplanned industrial and urban development in highly exposed coastal and riverine areas, which places critical infrastructure and industrial hubs in zones with a high probability of disasters (Djalante & Thomalla 2012; Fuady et al. 2025).

**TABLE 1:** Disaster event frequency and economic losses in Indonesia (2000–2016) after Meilano et al. (2022).

Hazards	*N* of events	Loss [T-IDR]	Loss per event [B-IDR]
Extreme weather events	4640	0.87	0.188
Flood	4229	74.25	17.557
Landslide	3783	20.60	5.445
Drought	1872	0.23	0.123
Forest fires	525	85.04	161.981
Earthquakes	396	120.91	305.328
Storm surge and abrasion	318	0.34	1.069
Volcanic eruption	111	19.94	179.640
Tsunami	44	43.38	985.909

Note: Please see the full reference list of this article Sakya, A.E., Hanifa, N.R., Shomim, A.F., Ratna, P.N., Oktaviani, A., Bisri, M.B. et al., 2026, ‘Enhancing geohazard-based natural hazards triggering technological accidents’ preparedness: A policy framework in Indonesian context’, *Jàmbá: Journal of Disaster Risk Studies* 18(1), a2062. https://doi.org/10.4102/jamba.v18i1.2062, for more information

T-IDR, Trillion Indonesian Rupiah; B-IDR, Billion Indonesian Rupiah.

Industrial regions such as Cilegon and Rokan Hilir, as well as areas of Kalimantan and Papua, are particularly vulnerable to such disruptions (Firman et al. 2011; Kurniawan et al. 2024; Lestari et al. 2021; Triyanti et al. 2023). Natural hazards triggering technological accidents studies in Indonesia have mostly focused on post-disaster response, while pre-disaster planning, industrial risk assessment and mitigation strategies for technological accidents triggered by geohazards have received minimal attention (Adri et al. 2020; Bisri 2016; Lestari et al. 2021; Ramada 2024). Despite these growing risks, Indonesia’s NATECH risk governance remains underdeveloped, characterised not only by limited regulations and insufficient sectoral awareness but also by the absence of an integrated framework linking geohazard assessment with industrial risk management (Adri et al. 2020; BNPB 2025; Lestari et al. 2021; Mulyasari & Nugraha 2024). At the regional level, the Association of Southeast Asian Nations (ASEAN) has yet to recognise NATECH as a regulatory priority (AHA Centre 2023). This absence reflects a broader gap in ASEAN’s disaster risk governance, where technological disasters triggered by natural hazards remain outside the scope of industrial standards and policy instruments. Several studies in Malaysia have addressed policy awareness regarding emerging technological disaster risks (Bin Mohd Zain 2022; Ramada 2024), critical infrastructure resilience to natural hazards (Yahaya et al. 2025), and coastal and maritime infrastructure (Batmanathan et al. 2025). However, these efforts remain limited to academic discourse and have not yet been translated into regulatory frameworks or industry standards. The need for a robust framework in Indonesia is further highlighted by successful international examples. Over the past two decades, increasing global awareness of NATECH risks has led to the integration of specific policies and operational protocols, such as the Seveso III Directive in Europe (EU Commission 2012) and Japan’s stringent post-Fukushima safety protocols (Krausmann et al. 2017). These frameworks demonstrate that effective NATECH mitigation requires a blend of top-down regulatory enforcement and bottom-up community resilience (Necci & Krausmann 2022). These international practices provide valuable insights for countries such as Indonesia, where industrial development is rapidly expanding into high-risk zones, and offer a strong foundation upon which to build a comprehensive national strategy (Lestari, Abdullah & Furqan 2023; Mulyasari & Nugraha 2024; Ramada 2024). Against this backdrop, Indonesia’s initial steps towards developing a NATECH framework, even if nascent, represent a critical move in bridging the regional gap. While ASEAN has yet to institutionalise NATECH standards, Indonesia’s efforts can serve as a catalyst for broader regional awareness and policy development. Establishing a national framework not only strengthens domestic resilience but also positions Indonesia as a frontrunner in shaping ASEAN’s future discourse on NATECH risk governance. Geohazards occurring in Indonesia, such as tsunamis, liquefaction and landslides, clearly require a tailored risk management approach and site-specific industrial safety standards (Al Farizi, Syamsidik & Mubarak 2023; Maraboutis, Poulimenou & Nikolau 2021). This approach requires contextual adaptation to industrial areas prone to geohazards, as well as the support of a relevant, integrated and comprehensive regulatory and institutional framework.

While Indonesia has made significant stride in managing primary geohazards, the secondary, cascading effects such as earthquake-induced chemical spills, tsunami-related energy infrastructure failures, or volcanic as interference with industrial cooling systems, remain a largely unmapped and under-regulated frontier. Without a dedicated NATECH risk assessment and integrated early warning system (EWS), localised geohazards threaten to escalate into large-scale technological disasters with long-term socio-economic and environmental consequences. Therefore, there is an urgent necessity to move beyond traditional disaster management and develop a specialised, data-driven NATECH governance framework tailored to the unique Indonesian geohazard landscape. This study aims to fill the gap in Indonesia’s NATECH risk governance by examining scholarly research, hazard mapping and regulatory frameworks.

We seek to answer the following questions:

- *How are NATECH events and associated hazards currently documented and managed in Indonesia?*
- *To what extent are NATECH risks integrated into national hazards and industrial safety policies?*
- *What governance strategies are necessary to enhance NATECH preparedness in Indonesia’s industrialising regions?*

By addressing these questions, this study aims to contribute to the development of a comprehensive NATECH risk mitigation framework tailored to Indonesia’s unique challenges.

The subsequent section outlines the methodology adopted to address these questions, followed by the key findings and a discussion of their implications. The article concludes by emphasising the urgent need for a comprehensive NATECH framework in Indonesia to manage the challenges posed by rapid national development coupled with the increasing frequency and severity of natural hazards across the country.

## Research methods and design

This study employs a multistage approach to assess NATECH risks in Indonesia combining quantitative bibliometric analysis, historical event reconstruction and spatial risk mapping to inform the development of a NATECH policy framework. The flow diagram of the methodology is presented in Figure 1-A1 in Appendix 1.

### Bibliometric literature study

This study began with a bibliometric analysis to map research trends and identify gaps in NATECH studies related to geohazards in Indonesia. Using VOSviewer (Jan van Eck & Waltman 2020) and the Preferred Reporting Items for Systematics reviews and Meta-Analyses (PRISMA) framework (Page et al. 2021), publications were retrieved from Scopus and Web of Science (WoS) in the period of 2007 until 2025 for further systematically reviewed from Scopus and Web of Science (2007–2025) were systematically reviewed. The search covered keywords on NATECH, cascading disasters, geohazards (earthquakes, tsunamis, floods, landslides, volcanic eruptions) and industrial risk. Figure 2-A1 in Appendix 1 depicts the structured Boolean logic and query results, following the PRISMA logic framework. The screening stage of Scopus and Web of Science database between 2007 and 2025 yielded 167 publications, of which 57 were duplicates. Further elaboration of the 110 articles resulted in only five articles significantly relevant to this study. Bibliometric analysis using VOSviewer produced co-occurrence revealing research patterns and country contributions. The results show limited attention to NATECH risks in Indonesia, highlighting the need for stronger geohazard mapping and policy frameworks tailored to local contexts. To strengthen this study, we added four articles to serve as a basis for the literature review we conducted prior to this study (Mesa-Gomes, Casal & Muñoz 2020; Naderpour et al. 2019; Suarez-Paba et al. 2019; Valente, Ricci & Cozzani 2025), and 38 publicly available sources, resulting in 47 articles were analysed for this study.

### Geohazard-driven natural hazards triggering technological accident events in Indonesia

Given the lack of standardised NATECH reporting system in Indonesia, a multisource approach, combining literature review, disaster databases, for example, National Agency for Disaster Management (BNPB), Emergency Events Database (EM-DAT), government reports and expert consultation, was used to compile a record of Indonesia’s geohazard-induced technological accidents. Events were identified through targeted keywords (see Figure 2-A1, Appendix 1) such as ‘earthquake-induced industrial accident’ and ‘tsunami-related technological disaster’. This process covers both major and lesser-known cases, including the 2004 Aceh tsunami-induced oil facility failure and the 2018 Palu liquefaction that hampered energy distribution and humanitarian logistics. Data were triangulated across independent sources to ensure reliability. Despite persistent issues such as under-reporting and non-standardised NATECH classification, this database establishes a baseline for understanding Indonesia’s exposure and institutional readiness.

### Geospatial hazard – Exposure mapping

The spatial intersection of geohazard and industrial assets was analysed using GIS-based overlay techniques (Burrough & McDonnell 1998) to identify where geohazards threaten industrial facilities. We utilised national datasets from the National Disaster Management Authority of Indonesia (BNPB) through the InaRISK web portal (http://inarisk.bnpb.go.id; BNPB 2025), National Seismic Source and Hazards Map for the National Center for Earthquake Studies (PuSGen 2017), Geological Agency (Badan Geologi 2023) and the Agency for Meteorology Climatology and Geophysics (BMKG 2025). Hazards that were analysed include seismic hazard probability map with occurrence period of 10% in 50 years for earthquakes, tsunami inundation map, active volcanoes, floods inundation, landslides and extreme weather.

Hazard datasets were standardised, classified into risk levels (low, medium, high) and spatially overlaid with economic zones’ location data to assess exposure. The resulting multihazard NATECH exposure maps visualise zones where technological systems intersect with natural hazard risks, supporting data-driven planning for disaster preparedness and mitigation.

### Framework development

The final phase involved the synthesis of empirical findings into a NATECH risk governance framework in Indonesia, drawing from bibliometric literature findings and geohazard NATECH exposure mapping. The framework addresses regulatory gaps and promotes integration of NATECH considerations into disaster risk reduction (DRR) and spatial planning policies.

Furthermore, a content analysis was undertaken to assess the extent to which these regulatory instruments: (1) explicitly reference NATECH or cascading disaster scenarios; (2) mandate the implementation of multihazard risk assessments within industrial sectors; and (3) establish provisions for cross-agency coordination and the integration of early warning systems. This analysis aims to identify regulatory gaps and opportunities for strengthening resilience against complex and interlinked disaster risks.

### Ethical considerations

This article followed all ethical standards for research without direct contact with human or animal subjects.

## Results

### Bibliometric literature study

The bibliometric review revealed that NATECH research in Indonesia remains limited, fragmented and often incorporated within broader DRR studies rather than addressed as a distinct research field. The existing literature reiterates that NATECH studies in Indonesia have focused more on post-disaster response, while pre-disaster planning, industrial risk assessment and mitigation strategies for technological accidents triggered by geohazards have received minimal attention. There is a marked deficit in studies concerning pre-disaster diagnostic tools, such as industrial fragility curves and predictive simulation for geohazard-induced technological failures. Literature reviews, generally conducted in developed industrial countries, emphasise case-based analysis, vulnerability assessment and simulation models for complex technological systems. These methodologies are still less applicable in Indonesia, where industrial characteristics and geohazards differ significantly. Thematic grouping of global studies reveals five dominant areas: (1) integration of NATECH into the broader disaster risk discourse, (2) mitigation and risk reduction strategies, (3) accident prevention mechanisms, (4) vulnerability and exposure analysis, and (5) governance and regulatory frameworks. While developed industrial economies prioritised high-fidelity simulation and complex system modelling, these methodologies show low translatability to the Indonesian context. The bibliometric mapping confirms a significant institutional decoupling between industrial disaster preparedness and national DRR frameworks. Industrial sectors such as oil and gas, mining and chemical processing, which are most vulnerable to cascading impacts, have not been systematically integrated into national hazard governance. Furthermore, cross-sectoral coordination between environmental, industrial and disaster management institutions remains limited, reducing the effectiveness of preparedness and early warning systems. Internationally recognised frameworks, such as the Organisation for Economic Cooperation and Development (OECD) Chemical Accident Programme (OECD 2023) and the EU Seveso Directive (EU Commission 2012) provide essential benchmarks for NATECH prevention and mitigation. However, amid the likelihood of cascade effects from every NATECH event (Girgin et al. 2019), Indonesia urgently needs an intervention that takes into account relevant institutional and regulatory frameworks in addition to regionally contextualised risk management and industrial safety standards.

Another key finding from the literature is the absence of measurable indicators for industrial resilience in hazard-prone areas. Data limitations and restricted access to industrial risk information hinder both academic and policy progress. Global guidance documents from OECD, United Nations Office for Disaster Risk Reduction (UNDRR) and the United Nations Office for the Coordination of Humanitarian Affairs (UNOCHA) provide valuable conceptual direction but lack operational metrics or legal enforcement mechanisms applicable to national contexts.

Overall, this review underscores the urgent need for a coherent NATECH governance framework in Indonesia, one that bridges scientific understanding, industrial regulation and disaster management practices. Strengthening institutional collaboration, establishing early warning linkages with high-risk industries and embedding NATECH indicators within DRR policy instruments are critical steps towards building industrial resilience in the face of geohazard-triggered disasters.

### Geohazard-driven natural hazards triggering technological accident events in Indonesia

Indonesia’s diverse geological setting exposes industrial systems to recurrent geohazard-induced technological disruptions. The documented cases illustrate how floods, landslides, land subsidence, volcanic eruptions, earthquakes and tsunamis have triggered cascading effects that disrupted industrial operations and revealed systemic regulatory and governance gaps.

#### Floods and extreme weather

Hydrometeorological disasters are increasing in frequency and severity, commonly disrupting oil, gas and manufacturing industries situated in lowland and coastal regions (BNPB 2025). In early 2024, extensive flooding in Riau’s Rokan Block, the country’s key oil-producing area, forced the temporary shutdown of over 300 wells, cutting production by 24 000 barrels per day and causing substantial economic losses (Setiawan 2024). Similarly, flooding at the PT Weda Bay Industrial Park in 2020 halted smelter operations because of inundated access routes (Yahya, Arief & Futaki 2020), while flash floods at the Lotte Chemical site in Cilegon in February 2024 caused worker fatalities and project delays (Putri 2024).

These incidents demonstrate a recurring NATECH pattern in which extreme weather events interrupt technological systems, exposing deficiencies in early warning systems, land-use regulation and hazard-resistant infrastructure design. Although smaller in scale compared to global events such as Hurricane Katrina, such disruptions highlight the growing economic and safety implications of unmitigated climate-related NATECH risks.

#### Landslides

Landslide-induced NATECH events increasingly threaten extractive industries in mountainous zones. In March 2025, intense rainfall triggered a landslide at the PT QMB New Energy Materials facility in the Indonesia Morowali Industrial Park (IMIP), Central Sulawesi, killing workers and halting operations. The collapse of a tailings storage area caused environmental risks and production losses estimated at $650 million (Da Costa 2025; Jong 2025). As rainfall intensity and land-use pressure grow, such events underscore the urgent need for robust site-specific risk assessments and regulatory oversight of tailings management in mining operations.

#### Land subsidence

Chronic land subsidence along Indonesia’s northern coastal corridor has caused structural damage to industrial facilities, pipelines and transportation infrastructure (Abidin et al. 2021; Harintaka et al. 2024). The phenomenon, exceeding 10 cm per year in parts of North Java, has intensified tidal flooding, affecting over 70 industrial zones and five special economic zones (SEZs). The Porong gas pipeline explosion (DetikNews 2006) exemplified a catastrophic NATECH scenario where infrastructure collapse was triggered by fire and inundation. Ongoing subsidence-related flooding in Semarang and Demak incurs annual losses exceeding IDR 2.6 trillion, with projections of up to IDR 82.7 trillion in long-term economic damage (Mahya, Kok & Van der Lelij 2021; Pratiwi et al. 2023).

#### Volcanic eruptions

Indonesia’s 127 active volcanoes frequently disrupt industrial and transport systems (Mahendra 2023). The 2017 Mount Agung eruption in Bali paralysed air traffic and fuel logistics, causing economic losses exceeding IDR-110 ($8.15 Million) (Antara 2017). The 2021 Mount Semeru eruption damaged mining facilities and power infrastructure in East Java, resulting in 51 deaths and $85m in losses (BNPB 2022). More recently, the 2025 Mount Ruang eruption in North Sulawesi disrupted maritime fuel and chemical transport through Bitung Port, leading to an estimated $65m in losses (Reliefweb 2024). Although these events did not trigger chemical releases, they reflect extended NATECH chains, where volcanic hazards indirectly compromise industrial continuity, logistics and energy systems (Pessina et al. 2021).

#### Earthquakes

Situated along multiple active faults, Indonesia faces recurrent seismic-induced industrial disruption. The 2018 Palu earthquake (Mw 7.5) and tsunami caused 4300 fatalities, destroyed fuel depots and small industries, and triggered secondary fires and leaks because of infrastructure collapse (Omira et al. 2019; Paulik et al. 2019; Widiyanto et al. 2019). Economic losses exceeded $911m (BNPB 2019). Similar vulnerabilities were observed following the 2021 Maluku (Mw 7.3) and 2024 Morowali (Mw 5.1) earthquakes, which disrupted ammonia storage and nickel smelter operations, respectively (Jong 2021; Ministry of Industry 2022). These events reveal the seismic fragility of Indonesia’s extractive industries and the lack of standardised protocols for structural integrity monitoring and rapid shutdown mechanisms in high-risk facilities.

#### Tsunamis

With over 95 000 km of coastline, Indonesia’s industrial assets face significant tsunami risk. The 2004 M9.2 Sumatra earthquake and Indian Ocean tsunami caused oil leaks and damage to fuel depots in Banda Aceh and Meulaboh, releasing 8000 kilolitres of oil (UNEP 2005). The 2018 Palu tsunami inflicted severe damage to ports, energy infrastructure and manufacturing zones, with total losses reaching IDR 18.8 trillion (BNPB 2019; World Bank 2018). Tsunami flow depths as low as 2 metres were sufficient to disable most industrial facilities because of inundation of control systems and short circuits (Cozzani et al. 2010).

Overall, these cases demonstrate that Indonesia’s industrial vulnerabilities to geohazards are both diverse and systemic. The recurrence of NATECH incidents across multiple hazard types reflects structural weaknesses in spatial planning, industrial zoning and emergency preparedness. Strengthening multihazard risk assessments, integrating NATECH considerations into SEZ planning and enforcing hazard-informed design standards are critical to minimising cascading losses and advancing industrial resilience.

### Risk assessment

The impacts of geohazards on industrial systems extend beyond direct physical damage, triggering cascading failures across infrastructure networks, supply chains and essential services. These systemic effects define the NATECH phenomenon, where natural hazards initiate technological disruptions with long-term socio-economic and environmental consequences (Gill & Malamud 2014; Krausmann et al. 2017). Comparative global events, such as Hurricane Katrina (2005) and the 2023 Turkey–Syria earthquake, demonstrate how geohazards can cause industrial shutdowns, energy supply interruptions and hazardous material releases, leading to multi-billion-dollar losses (Cruz & Krausmann 2009; World Bank 2023). In Indonesia, similar conditions prevail in regions where rapid industrialisation overlaps with high seismic, volcanic and hydrometeorological exposure zones. The geospatial hazard-exposure overlay analysis reveals extensive overlaps between high-hazards zones of earthquakes, tsunamis, volcanic eruption, landslides, extreme-weather and flood-prone places and major industrial hubs (Figure 1a to f). Furthermore, Figure 2 summarises the multihazard exposures across Indonesia’s SEZs.

**FIGURE 1:**
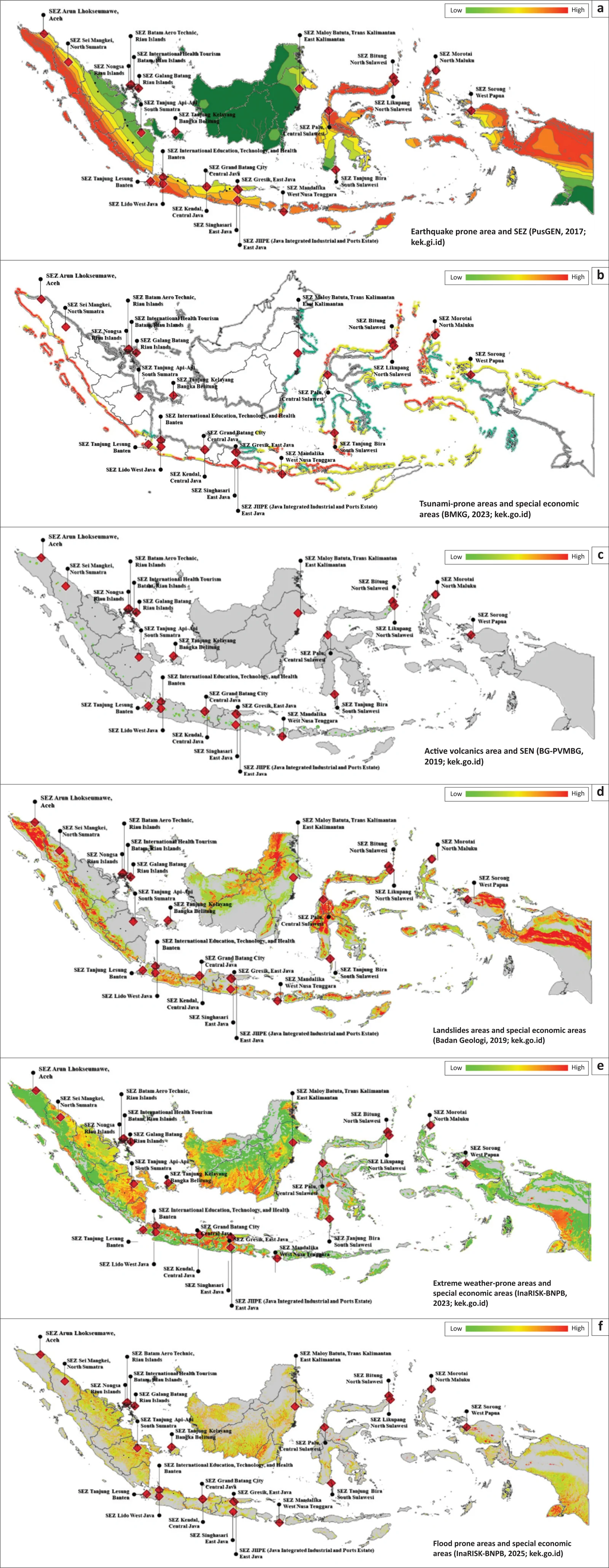
Locations of special economic zones (SEZ) in Indonesia overlaid over potential hazards such as (a) earthquakes, (b) tsunamis, (c) active volcanoes, (d) landslides, (e) extreme-weather, and (f) flood-prone places.

**FIGURE 2:**
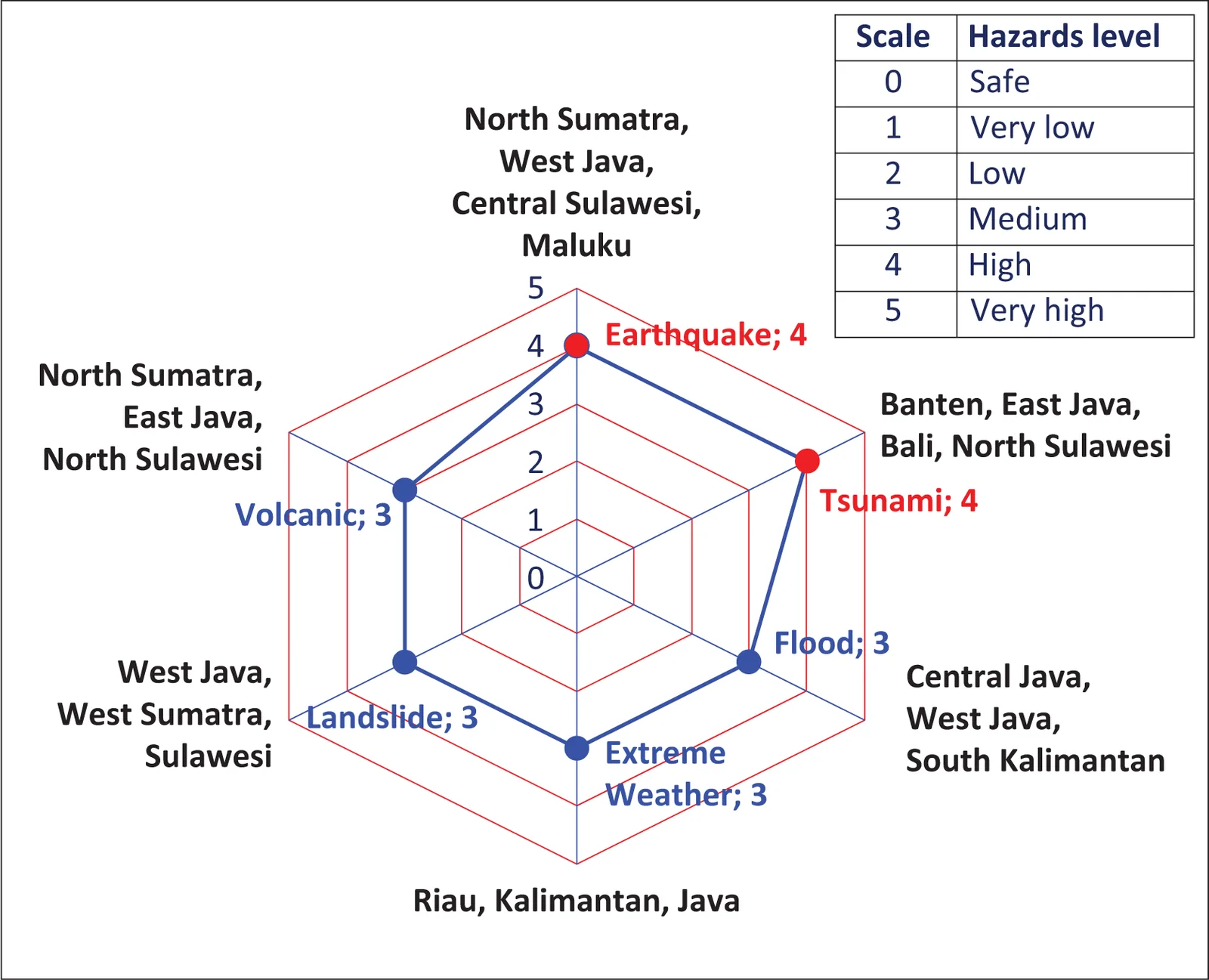
A summary of multihazard exposure across Indonesia’s special economic zones.

Earthquakes and tsunamis pose the highest risks, while floods and extreme weather have recurrent impacts on industrial operations. The chart highlights that nearly all SEZs experience exposure to more than one geohazard type, underscoring the need for integrated multihazard assessment and risk-informed planning in industrial development. Approximately, one-third of SEZs are situated within moderate to high earthquake-risk areas, particularly in North Sumatra, West Java, Central Sulawesi and Maluku, while coastal SEZs in Banten, East Java, Bali and North Sulawesi are directly exposed to tsunami threats. Flooding and extreme weather hazards pose the most frequent risks, affecting SEZs in Central and West Java, South Kalimantan and Riau. Volcanic and landslide hazards, although less frequent, threaten industrial logistics and transport corridors in mountainous regions such as West Sumatra and Sulawesi. These findings demonstrate that Indonesia’s industrial development is increasingly concentrated in multihazard environments, thereby amplifying systemic vulnerability and cascading risk potential. The analysis demonstrates that nearly all SEZs are multihazard-exposed, amplifying the potential for cascading failures that may disrupt national supply chains and essential infrastructure. Industrial shutdowns, material leakage or power interruptions in these zones can propagate rapidly through interconnected systems, compounding economic losses. Mitigation strategies should prioritise early warning integration, risk-informed land-use planning and hazard-resilient design standards. Industrial operators in high-risk areas must incorporate real-time monitoring, contingency planning and cross-sectoral communication protocols to minimise operational downtime and prevent technological accidents.

In summary, this assessment reveals that Indonesia’s current industrial expansion strategy lacks comprehensive hazard-informed planning. The clustering of critical industries within seismically and hydro-meteorologically active zones heightens the probability of NATECH events. Proactive risk governance, anchored in integrated hazard assessment and institutional accountability, is therefore essential to prevent cascading industrial disasters. The next section examines the existing regulatory landscape and identifies critical policy gaps in addressing NATECH governance in Indonesia.

### Regulatory assessment and framework development

Indonesia’s regulatory landscape for managing NATECH risks is broad but fragmented, with relevant provisions dispersed across disaster management, environmental protection, industrial safety, spatial planning and early warning systems. Although Indonesia has established a strong foundation in multihazard disaster governance, these frameworks do not yet integrate natural hazard triggers with technological accident prevention in a systematic manner. This fragmentation reflects a broader structural limitation in disaster risk governance, where hazard-specific regulations and industrial safety standards operate in parallel rather than within an integrated multihazard and cascading risk framework (OECD 2023; UNDRR 2022).

The subsequent subsections present the findings of: (1) an assessment of pertinent Indonesian regulations (Table 1-A1 in Appendix 1), (2) a comparative analysis against international best practices (Table 2-A1 in Appendix 1), and (3) a synthesis of identified deficiencies via the NATECH gap matrix. This analysis is conducted to prioritise regulatory strengthening and address the previously identified gaps.

#### Review of existing Indonesian regulations

Indonesia possesses an extensive regulatory foundation relevant to hazard governance and industrial risk management. As summarised in Table 1-A1 in Appendix 1, key instruments include the national Disaster Management Law (UU 24/2007), the Meteorology, Climatology and Geophysics Law (UU 31/2009), environmental protection regulations (PP 22/2021), hazardous materials controls regulation (Permenperin 87/2009), environmental risk assessments regulation (PermenLHK P.15/2019), occupational safety regulations and macro-scale spatial planning or detailed spatial planning frameworks (RTRW or RDTR) that designate hazard-prone zones.

Despite this breadth, the regulatory review reveals the following important limitations:

**No explicit mandate for natural hazards triggering technological accidents risk assessment:** Environment Impact Assessment (AMDAL), Occupational Health and Safety (K3) and industrial permits do not require modelling of external hazard-triggered failure scenarios (e.g. earthquake-induced tank rupture, tsunami-driven chemical release). This gap indicates that current risk assessment frameworks remain predominantly hazard-specific that is facility-based, without incorporating cascading multihazard interactions, which are central to NATECH risk characterisation (Krausmann et al. 2017; OECD 2023).

**Sectoral fragmentation persists:** National Disaster Management Agency (BNPB), Ministry of Environment and Forestry (KLHK), Agency for Meteorology, Climatology and Geophysics (BMKG), Ministry of Industry (Kemenperin) and SEZ authorities operate largely independently, with no shared risk database, joint inspections and harmonised emergency protocols for high-risk industrial facilities. Such institutional fragmentation reduces the effectiveness of early warning systems and limits the ability to implement coordinated responses to disasters (Triyanti et al. 2023; UNDRR 2022).

**Weak integration of hazard information into industrial permitting:** Although hazard maps exist under Regional Spatial Planning/Spatial Detail Planning (RTRW/RDTR), they are not systematically linked to SEZ approval or high-risk industrial siting decisions. This disconnect between spatial planning and industrial licensing reflects a critical regulatory and governance gap, where hazard exposure is not adequately translated into regulatory control over industrial development (Djalante & Thomalla 2012; OECD 2023). Thus, Table 1-A1 in Appendix 1 indicates that Indonesia’s regulatory system remains primarily hazard-oriented, not NATECH-oriented, and does not yet support the type of cascading risk governance required in high-exposure industrial corridors, which reemphasises findings from previous studies (Lestari et al. 2018). Furthermore, to understand what Indonesia lacks, we benchmarked against leading global NATECH governance frameworks, including the OECD Chemical Accident Programme (OECD 2023), the European Union’s Seveso III Directive (EU Commission 2012), UN Environment/OCHA Join Unit (2017), and United Nations Environment Programme Awareness and Preparedness for Emergency at Local Level (UNEP APELL) (UNEP 2015).

These frameworks share a broad set of core principles, including:

- Mandatory Environmental Impact Assessment (EIA) external hazard impact modelling for industrial facilities.
- Integration of early warning systems with automated industrial response.
- Standardised NATECH preparedness indicators for compliance and auditing.
- Integrated incident reporting mechanisms to support learning and accountability.
- Formal multi-agency coordination, including joint inspections.
- Hazard-based land-use planning for industrial sites.

These features, central to global best practice, are largely absent from Indonesia’s current regulatory system as documented in Table 1-A1 in Appendix 1.

#### The synthesis of the natural hazards triggering technological accidents gap

By comparing existing Indonesian regulations (Table 1-A1, Appendix 1) with global best practices, a clear set of regulatory gaps emerges. These gaps are not limited to technical issues but reflect structural governance deficits, most importantly, the absence of a dedicated NATECH policy instrument and the lack of integration between hazard monitoring authorities, environmental regulators and industrial safety bodies.

A consolidated overview of these gaps is presented in Table 2-A1 in Appendix 1, which identifies 10 critical NATECH governance components, contrasts international best practices with Indonesian provisions and highlights the implications for national industrial resilience. The combined insights from Table 1-A1 and Table 2-A1 (Appendix 1) illustrate the following coherent pattern. Despite Indonesia’s robust foundation in hazard governance, environmental protection and industrial safety, there is currently no integration of these systems to tackle NATECH-specific risks. This fragmentation results in structural vulnerabilities for industrial facilities, particularly in SEZs, where exposure to earthquakes, tsunamis, floods and extreme weather is high. This condition reflects what is often described in disaster governance literature as a ‘policy gap between risk knowledge and regulatory implementation’, where hazard information exists but is not operationalised within binding regulatory instruments (UNDRR 2022; OECD 2023).

The following are the strategic implications:

- Regulatory modernisation is necessary.
  Indonesia requires a dedicated, cross-sectoral NATECH regulatory instrument or at least mandatory NATECH assessment embedded into AMDAL (EIA) and industrial permitting.
- Institutional integration must be strengthened.
  Early warning system (BMKG), DRR (BNPB), Environment (KLHK, Ministry of Environment and Forestry), Industry (Kemenperin, Ministry of Industry), Labour (Kemnaker, Ministry of Labour) need a shared NATECH governance platform supported by joint inspections and integrated risk databases.
- Industrial safety planning must shift from reactive to anticipatory.
  Adopting international best practices, such as external hazard scenario modelling, automated shutdown protocols and standardised preparedness indicators, will significantly enhance industrial resilience.

## Discussion

The findings of this study reveal that NATECH risks in Indonesia emerge from a convergence of three major dynamics: (1) increasing geohazard frequency (BNPB 2024), (2) rapid industrial expansion in hazard-prone areas (KEK 2025), and (3) the absence of a coherent regulatory framework to prevent or mitigate hazard-triggered technological disasters (Table 1-A1, Appendix 1). This discussion encapsulates empirical findings, assesses their theoretical and policy ramifications and delineates strategic directions for strengthening NATECH governance in Indonesia.

### Interpretation of key findings

The bibliometric analysis (‘Bibliometric literature study’ in the ‘Results’ section) demonstrates that research on NATECH in Indonesia remains sparse, fragmented and largely descriptive. Despite the country’s high exposure to multihazard risks, there is no consolidated body of knowledge that systematically integrates geohazard science with technological risk management. This gap in the academic landscape is mirrored in documented NATECH events (‘Geohazard-driven natural hazards triggering technological accident events in Indonesia’ section), where floods, earthquakes, tsunamis, volcanic eruptions and landslides have repeatedly disrupted industrial operations, causing casualties, environmental contamination and major economic losses. These incidents highlight the systemic vulnerability of industrial zones, especially SEZs situated along coastlines and tectonic corridors. The ‘Risk assessment’ section further shows that many SEZs are located in areas of overlapping hazard exposure. Earthquakes and tsunamis represent the most acute threats, while floods and extreme weather events create recurrent disruptive pressures. Industrial facilities within these zones often lack standardised risk assessments that incorporate cascading geohazard–technological interactions. This situation aligns with recent findings regarding the so-called ‘compound and cascading risk environments’, the tendency in which multiple hazards interact with exposed systems and amplify the risk scale beyond the assumption of a single hazard (De Ruiter et al. 2019; Sulfikkar Ahamed et al. 2023).

‘Regulatory assessment and framework development’ section reveals that Indonesia’s regulatory framework, although comprehensive in disaster management and environmental protection, does not yet incorporate NATECH-specific requirements. Table 1-A1 in Appendix 1 shows that existing regulations address hazards and technological risks separately. Table 2-A1 in Appendix 1 synthesises the gaps, showing that Indonesia lacks mandatory external hazard scenario modelling, industrial EWS integration, cross-sector inspections, unified incident reporting and standardised preparedness indicators. Collectively, these findings indicate that Indonesia’s current approach to managing NATECH risk is *reactive rather than anticipatory, sectoral rather than integrated and hazard-focused rather than cascade-oriented* (De Ruiter et al. 2019; Sulfikkar Ahamed et al. 2023; UNDRR 2022).

### Theoretical and practical implications

From a theoretical perspective, this study reinforces the argument that NATECH risks must be conceptualised as cross-boundary phenomena, transcending traditional divides between natural hazard science and technological risk management. Indonesia’s experience confirms the inadequacy of siloed disaster and industrial governance models in multihazard environments. The findings align with global best practices that emphasise the importance of systems thinking, multihazard modelling and cascade analysis.

Practically, the results demonstrate that industrial safety in geohazard-prone regions cannot rely solely on structural engineering or facility-level emergency planning. Instead, industrial resilience requires alignment between hazard monitoring agencies, environmental regulators, industrial authorities, spatial planners and SEZ administrators (OECD 2023; OECD & UN-HABITAT 2026). Without such coordination, even well-engineered facilities remain vulnerable to cascading failures triggered by earthquakes, tsunamis or floods.

### Policy implications

The gap analysis (Table 2-A1, Appendix 1) provides a strong foundation for policy reform. Four policy implications emerge:

- Need for a dedicated NATECH regulatory instrument: Indonesia lacks a legally binding mechanism that mandates NATECH risk assessment for high-hazard industrial activities. Embedding NATECH requirements into AMDAL/EIA, SEZ permitting and industrial safety regulations is essential.
- Integration of early warning systems into industrial operations: the earthquake, tsunami, and extreme weather warnings produced by the Agency for Meteorology Climatology and Geophysics (BMKG) must be linked to industrial shutdown protocols and automated alerts, similar to Japanese and EU practices.
- Institutional coordination and shared risk databases: Current institutional fragmentation prevents holistic risk governance. A multiagency NATECH task force or integrated authority could bridge these gaps.
- Strengthened spatial planning for industrial siting: Hazard maps must be systematically incorporated into land-use decisions for industrial development, especially in SEZs.

These policy directions align with international frameworks and reflect the minimum requirements for effective NATECH governance (Table 2-A1, Appendix 1)

### Limitations and future research

This study is constrained by the limited availability of documented NATECH incidents in Indonesia, reflecting under-reporting at national and industrial levels.

Future research should prioritise, among others:

- Development of a national NATECH database.
- Facility-level hazard-triggered vulnerability modelling.
- Cost–benefit assessments of NATECH mitigation measures.
- Integration of climate change scenarios (extreme rainfall, sea-level rise, compound hazards).

These areas will strengthen the empirical foundation for NATECH policy development. Furthermore, the above discussion demonstrates that Indonesia is at a critical juncture. While exposure to geohazards will continue to increase, industrial expansion into hazard-prone areas is accelerating.

Without systemic regulatory reform, cascading NATECH events are likely to become more frequent and severe. By aligning national regulations with international best practices and adopting integrated, multihazard approaches, Indonesia can significantly enhance the safety and resilience of its industrial systems.

### Policy recommendations

Based on the gaps identified, five priority policy recommendations are proposed:

- Develop a dedicated national NATECH regulation or integrated guideline: This should embed NATECH risk assessment into AMDAL, SEZ permitting, hazard zoning and industrial safety regulations.
- Mandate external hazard-triggered scenario analysis for high-risk industries: Models should include seismic-induced tank failures, tsunami-driven chemical releases, flood-triggered power outages and extreme weather-induced structural failures.
- Integrate BMKG’s early warning systems with industrial emergency response protocols: Automatic shutdown mechanisms, remote valves, protective barriers and communication protocols must activate based on real-time hazard alerts. Linking early warning systems to industrial operations is essential to minimise escalation from hazard events into technological disasters.
- Establish standardised NATECH preparedness indicators for SEZs and high-risk sectors: These indicators should guide compliance auditing, monitoring and performance scoring, similar to Seveso III and OECD benchmarks.
- Strengthen cross-sector institutional coordination: A national multiagency task force involving BNPB, KLHK, BMKG, Kemenperin, Kemnaker and SEZ authorities should oversee inspections, information sharing and NATECH incident reporting.

### Implications for practice and governance

Implementing NATECH-sensitive regulations will strengthen industrial resilience and reduce losses during future disasters. Integrating hazard information into industrial licensing and spatial planning will help prevent dangerous clustering of high-risk facilities in vulnerable zones.

Moreover, linking early warning systems to industrial operations enhances preparedness and minimises cascade effects during earthquake or tsunami emergencies. Collectively, these measures will yield multilevel benefits, strengthening community safety, protecting supply chains and safeguarding national economic stability.

### Future research directions

The study identifies several areas where further research is essential:

- Development of Indonesia’s first national NATECH incident database, integrating government and industry reporting.
- Quantitative modelling of multihazard cascade scenarios for petroleum, chemical, mining and energy facilities.
- Cost–benefit analysis of NATECH mitigation at the facility and SEZ level.
- Integration of climate change projections into NATECH risk, especially regarding extreme rainfall, sea-level rise and compound hazards.
- Community–industry interface studies to support APELL-type coordination in SEZs.

Indonesia stands at a strategic crossroads. While its industrial development is accelerating, geohazard exposure continues to intensify. Without targeted reforms, the likelihood and severity of NATECH events will increase. By adopting integrated, multihazard-informed and internationally aligned governance frameworks, Indonesia can significantly enhance the resilience, safety and sustainability of its industries. The findings and recommendations presented in this study offer a practical roadmap for achieving that goal.

## Conclusion

This study highlights the growing importance of addressing NATECH risks in Indonesia, a country characterised by intense geohazard activity and rapid industrial expansion. Through a comprehensive literature review, case-based assessment, spatial risk mapping and regulatory analysis, the study reveals that Indonesia’s industrial landscape, particularly within SEZs, faces significant exposure to earthquakes, tsunamis, floods, volcanic eruptions, landslides and extreme weather. These hazards have already triggered multiple industrial disruptions and cascading impacts, resulting in economic losses, environmental contamination and safety risks. Despite having a robust foundation in disaster management and environmental governance, Indonesia’s regulatory system remains fragmented, sectoral and not yet NATECH-informed. Existing regulations address natural hazards and technological risks separately, with no binding requirements for hazard-triggered scenario modelling, integrated early warning systems or multiagency oversight. Benchmarking with international best practices (OECD, Seveso III, UNDRR, UNEP) confirms the absence of essential NATECH governance components, as summarised in the NATECH Gap Matrix. Overall, the study concludes that Indonesia urgently needs a coherent, multisectoral and anticipatory regulatory framework that integrates geohazard science with industrial risk management to effectively reduce cascading disaster risks.

## Figures and Tables

**FIGURE 1-A1:**
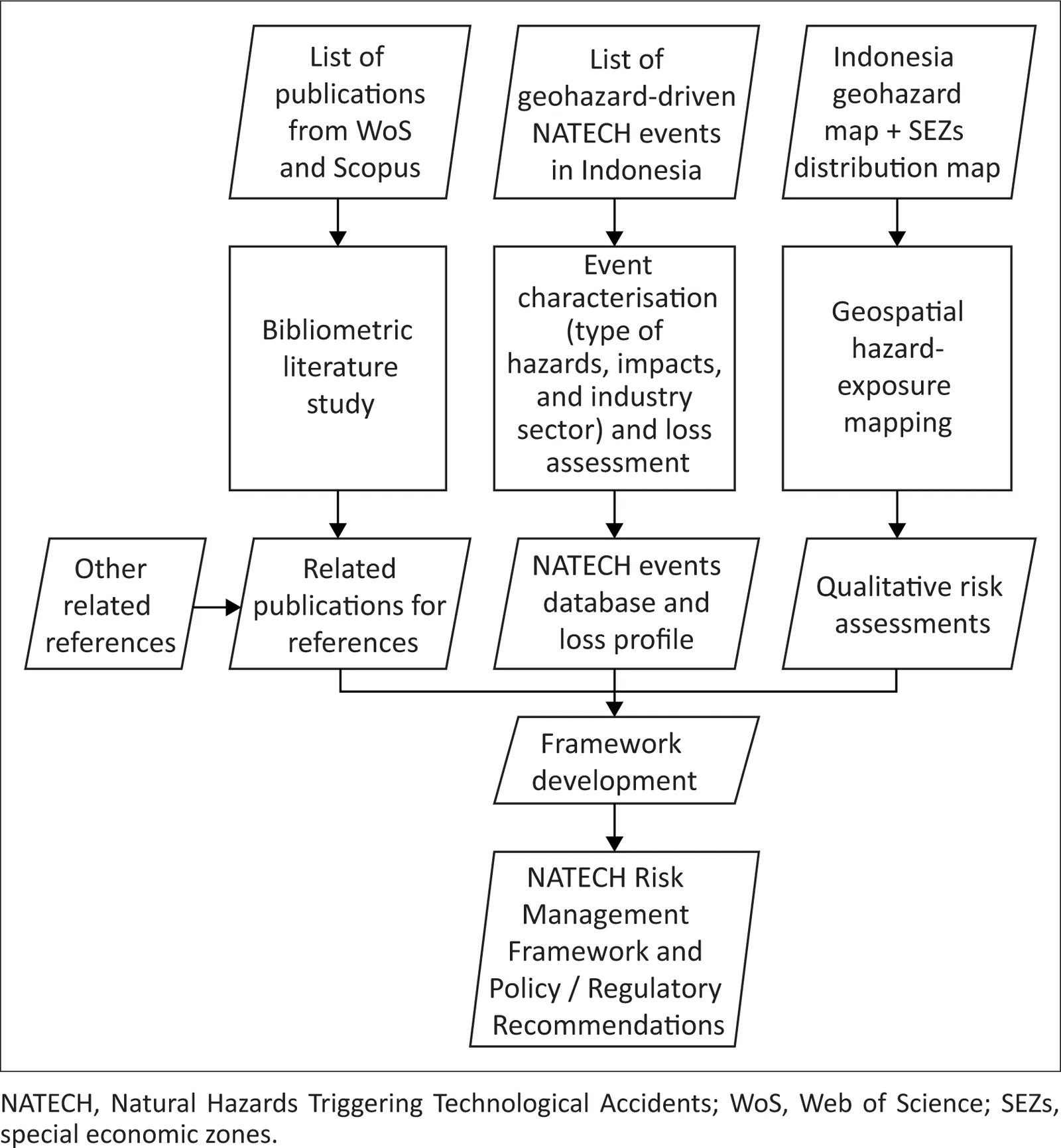
Methodological flowchart for the analysis of geohazard-driven Natural Hazards Triggering Technological Accidents events in Indonesia, including literature review, event characterisation, geospatial exposure mapping, risk assessment and framework development leading to risk management and policy recommendations.

**FIGURE 2-A1:**
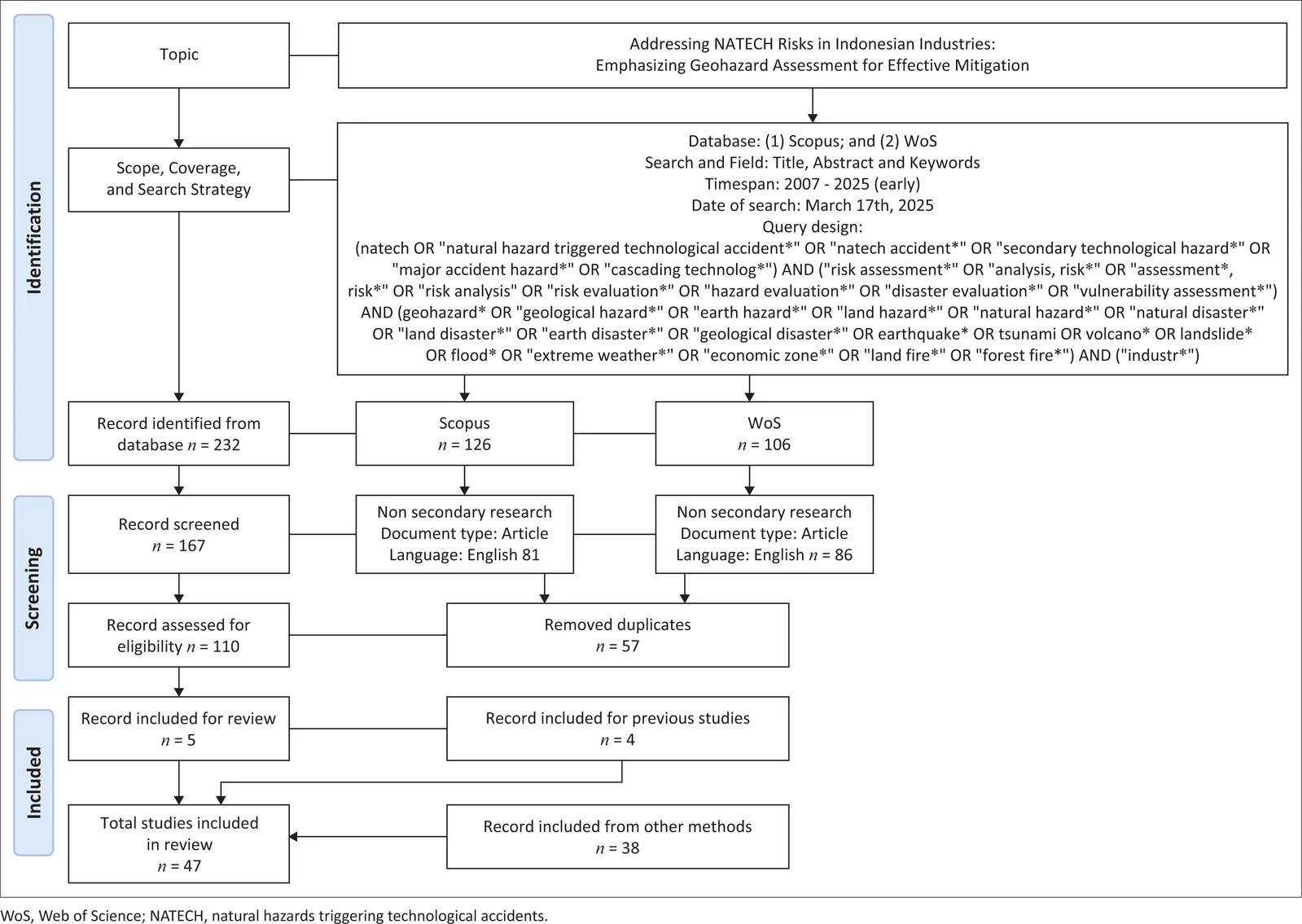
The Preferred Reporting Items for Systematic reviews and Meta-Analyses table that illustrates the bibliometric literature study process in this study.

## Appendix 1

**TABLE 1-A1:** List of Indonesian regulations related to Natural Hazards Triggering Technological Accidents risk management.

Hierarchy of regulation	Regulation / instrument	Scope / key provisions	Relevance to NATECH
Laws (UU)	UU No. 24/2007 – Disaster Management Law	National DRR framework; mandates risk analysis, preparedness and early warning	Covers natural hazards, but no technological cascade requirements
	UU No. 31/2009 – Meteorology, Climatology, and Geophysics Law	Establishes BMKG; mandates hazard monitoring and early warning	Enables hazard alerts but not linked to industrial protocols
Government regulations (PP)	PP No. 21/2008 – Disaster Management Implementation	Operational guidelines for multihazard assessment	Does not mandate industry-specific hazard-triggered risk analysis
	PP No. 22/2021 – Environmental Protection & Management	AMDAL requires environmental risks assessment	Potential entry point, but no explicit NATECH scenario requirements
	PP No. 40/2021 – Special Economic Zones	Location and spatial planning	Does not address and consider potential hazards zones mapping
Ministerial regulations (Permen)	Permenperin No. 87/2009 – Hazardous Materials (B3)	Regulates B3 management	Relevant to spill/leak risks but excludes natural hazard triggers
	PermenLHK P.15/2019 – Environmental Risk Assessment	Environmental risk appraisal standards	Geohazards included indirectly; no cascading scenario analysis
	PermenPUPR No. 21/2019 – Infrastructure Disaster Management	Construction management safety and infrastructure resilience guidelines	Does not address industrial NATECH vulnerabilities
	Permenhub PM 60/2019 – Port/Maritime Safety	Maritime hazard and safety management	Relevant for tsunami/storm-induced industrial accidents
	Permenaker No. 5/2018 – Safety Standards	Occupational safety standards	Industrial accidents only; excludes natural hazard triggers
	No. 11/2023 – Safety standards	Regulated safety standards in confined space	Industrial accidents only; excludes natural hazard triggers
Agency regulations	Perka BNPB No. 4/2008 – Disaster Planning	Hazard data integration into DRR planning	No industrial cascade risk requirements
	PerBMKG No. 9/2010 – Procedure Extreme Weather Information	Extreme Weather EWS protocol	Not integrated into industrial shutdown protocols
	PerBMKG No. 9/2019 – EWS Extreme Climate	Extreme climate EWS Protocol	Not integrated into industrial shutdown protocols
	PerBMKG No. 9/2022 – Provision on the dissemination of EWS Extreme Weather	Effectivity of extreme weather dissemination	Not integrated into industrial shutdown protocols
	PerBMKG No. 6/2023 – Air Quality EWS Protocols	Air quality EWS Protocol	Not integrated into industrial shutdown protocols
Spatial Planning & SEZ Regulations	PermenATPBPN No. 5. 2022 – SOP on Spatial Planning	Land-use, zoning, hazard-prone area designation	Hazard maps not systematically linked to industrial licensing and geohazards

NATECH, Natural Hazards Triggering Technological Accidents; DRR, disaster risk reduction; BMKG, Agency for Meteorology Climatology and Geophysics; AMDAL, Analisis Mengenai Dampak Lingkungan [*Environmental Impact Assessment*]; EWS, Early Warning System; SEZ, special economic zone.

**TABLE 2-A1:** Natural Hazards Triggering Technological Accidents Gap Matrix: Comparison of international best practices and Indonesian regulatory provisions.

No	NATECH governance component	International best practices	Current Indonesian regulations (Table 1-A1)	Gap identified	Implications for Indonesia
1	External hazard-triggered scenario analysis	Required under OECD, Seveso III, Japan (mandatory multihazard scenario modelling)	Not required in AMDAL, K3 or industrial permits	No legal mandate for NATECH scenario assessment	Industrial facilities in seismic/tsunami zones remain unprepared for cascading failures
2	Integration of early warning systems (EWS) with industrial operations	EWS linked to automatic alarms, shutdowns (Japan, EU)	BMKG issues alerts but no linkage to industrial responses	EWS not embedded into industrial protocols	Delayed response during earthquakes, tsunamis, extreme weather may lead to chemical releases
3	Mandatory NATECH preparedness indicators	OECD/Seveso require measurable safety performance indicators	PROPER and K3 lack NATECH-specific metrics	No standardised indicators for industry	Varied preparedness quality; difficult for government to enforce minimum standards
4	Unified incident reporting for NATECH events	Mandatory reporting to central authority (OECD, EU)	Indonesia lacks NATECH reporting mechanism	No national NATECH database	Limited monitoring, weak learning cycle; recurring incidents remain undocumented
5	Multiagency coordination and joint inspections	Integrated inspections: industry + environment + emergency authority	Sectoral silos: KLHK, BNPB, Kemenperin, BMKG operate independently	No formal cross-sector inspection framework	Fragmented oversight; gaps in industrial safety audits regarding geohazards
6	Land-use planning linked to industrial permitting	Seveso LUP guidelines require hazard-informed siting	RTRW/RDTR hazard maps not mandatory for industrial permits or SEZ approval	Weak integration of hazard zoning into permits	SEZs and industries often located in high-risk zones without mitigation
7	Community–industry emergency coordination	APELL & Seveso require community inclusion and public information	Emergency planning operator-driven; limited public involvement	Lack of community engagement in NATECH preparedness	Low community awareness, slower evacuation, higher fatalities during disasters
8	Infrastructure and equipment resilience standards	EU require seismic design, flood protection, elevated storage, redundancy	Indonesian industrial standards do not include multi-hazard resilience	No hazard-specific infrastructure requirements	Higher probability of structural failure during geohazards
9	Post-disaster environmental and industrial assessment	FEAT 2.0 mandates rapid environmental and chemical risk assessment	Not standardised in BNPB or KLHK protocols	No rapid post-disaster industrial risk assessment guidelines	Chemical spills, contamination, and fires may go undetected after disasters
10	NATECH-specific regulatory instrument	EU Seveso III dedicated industrial hazard regulation	Indonesia has no dedicated NATECH or major-accident regulation	NATECH unrecognised as a regulatory category	System remains reactive rather than preventive; no clear accountability

NATECH, Natural Hazards Triggering Technological Accidents; OECD, Organisation for Economic Cooperation and Development; DRR, disaster risk reduction; BMKG, Agency for Meteorology Climatology and Geophysics; AMDAL, Analisis Mengenai Dampak Lingkungan [*Environmental Impact Assessment*]; KLHK, Kementerian Lingkungan Hidup dan Kehutanan [*Ministry of Environment and Forestry*]; BNPB, Badan Nasional Penanggulangan Bencana [*National Disaster Management Agency*]; BMKG, Badan Meteorologi, Klimatologi, dan Geofisika [*The Agency for Meteorology, Climatology, and Geophysics*]; RTRW, Rencana Tata Ruang Wilayah [*Regional Spatial Plan*]; RDTR, Rencana Detail Tata Ruang [*Detailed Spatial Plan*]; EWS, Early Warning System.
